# Correlations between Traditional and Nontraditional Indicators of Adiposity, Inflammation, and Monocyte Subtypes in Patients with Stable Coronary Artery Disease

**DOI:** 10.1155/2019/3139278

**Published:** 2019-07-03

**Authors:** Silvia Bueno Garofallo, Vera Lucia Portal, Melissa Medeiros Markoski, Lucinara Dadda Dias, Alexandre Schaan de Quadrosa, Aline Marcadenti

**Affiliations:** ^1^Graduate Program in Health Sciences (Cardiology), Institute of Cardiology of Rio Grande do Sul/University Foundation of Cardiology (IC/FUC), Princesa Isabel Avenue, 395, Porto Alegre, Rio Grande do Sul 90040-371, Brazil; ^2^Graduate Program in Nutrition Sciences, Federal University of Health Sciences of Porto Alegre (UFCSPA), Sarmento Leite Street, 245, Porto Alegre, Rio Grande do Sul 90050-170, Brazil; ^3^Institute of Research,Coracao Hospital (HCor), Abílio Soares Street, 250, São Paulo, São Paulo 04004-05, Brazil

## Abstract

**Background:**

Recruitment of monocytes and low-grade inflammation process are both involved in obesity and in atherosclerosis. Thus, the aim of this study was to evaluate the correlation among indicators of adiposity, monocyte subtypes, and inflammatory markers in patients with stable coronary artery disease (CAD).

**Methods:**

This was a cross-sectional study including 97 patients with stable CAD aged >40 years. Traditional anthropometric indicators of adiposity (body mass index (BMI); waist, hip, and neck circumferences; and waist-hip ratio) and nontraditional anthropometric indicators of adiposity (lipid accumulation product index (LAP), visceral adiposity index (VAI), and deep-abdominal-adipose-tissue index (DAAT)) were determined. Immunoprecipitation, turbidimetry, coagulometric method, and CBA were used for the evaluation of inflammatory markers (hs-CRP, IL-2, IL-4, IL-6, IL-10, and INF-*γ*). Monocyte subtypes were identified by flow cytometry and defined as CD14++ CD16− (Mon1), CD14++ CD16+ (Mon2), and CD14+ CD16++ (Mon3). Pearson's correlation coefficient and adjusted partial correlation were calculated.

**Results:**

Monocyte subtypes were correlated with inflammation regardless of nutritional status according to BMI. In overweight individuals, LAP was correlated with IL-4 and fibrinogen (*P* < 0.01 and *P* < 0.05, respectively) and VAI with IL-4 (*P* < 0.05). In obese patients, the BMI, waist, neck, and hip circumferences, and DAAT were correlated with IL-6 (*P* < 0.05), regardless of age and sex. The hip circumference was correlated positively with Mon1 (*r* = 0.40, *P* = 0.007) and negatively with Mon3 (*r* = −0.35, *P* = 0.02) in obese subjects.

**Conclusion:**

Monocyte subtypes are correlated with inflammation in patients with stable CAD independently of BMI, whereas traditional and nontraditional indicators of adiposity are correlated differently with inflammatory markers and monocytes, according to the nutritional status.

## 1. Introduction

In atherosclerosis and obesity, the recruitment of monocytes and the infiltration of macrophages into the endothelium and adipose tissue [1], respectively, are pathophysiological mechanisms of these two distinct conditions. The low-grade inflammation resulting from this process leads to a dysfunctional state, with an increased risk for cardiovascular events [2]. However, the mechanisms connecting atherosclerosis and obesity are not completely understood although both have been linked in observational studies [1].

Increased adiposity, particularly visceral obesity, is associated with subclinical inflammation [3] and risk of coronary artery disease (CAD) [4]. Hence, indicators of adiposity to estimate the visceral adipose tissue (VAT) and its metabolic activity have been proposed, such as the lipid accumulation product (LAP) index [5], visceral adiposity index (VAI) [6], and deep-abdominal-adipose-tissue (DAAT) index [7]. The association of these indicators with atherosclerosis [8] and low-grade inflammation [9] has already been demonstrated; as such, these indicators would complement the use of body mass index (BMI), waist, hip, and neck circumferences, and waist-hip ratio to identify cardiovascular risk [10–12].

Monocytes act on the expression of inflammatory markers and are strongly involved in all stages of atherosclerosis [13–16]. Rogacev et al. [17] observed a positive relationship between monocyte subtypes and traditional indicators of adiposity in high cardiovascular risk patients. In humans, these cells are divided into three subtypes as follows [14, 17]: Mon1 (CD14+ CD16+), Mon2 (CD14++ CD16+), and Mon3 (CD14+ CD16++).

It has been shown that the concentrations of each monocyte subtype may vary according to the clinical manifestation of CAD [14, 15]. Higher levels of Mon2, which are considered to represent the most proinflammatory of the monocyte subtypes, followed by Mon1, increase the risk for cardiovascular events [14, 17]; however, no changes in the counts of either Mon2 or Mon3 were observed in patients with stable CAD compared to healthy individuals [14]. In addition, hyperlipidaemia [13] and changes in the renin-angiotensin system [18], both of which are commonly present in obesity [19], seem to potentiate the migration of these cells.

However, the relationship between nontraditional indicators of adiposity and the monocyte subtypes has not yet been established. Given the relationship between adipose tissue and subclinical inflammation and between monocyte concentration and inflammatory marker expression and considering the importance of this triad for the development and progression of CAD, the aim of this study was to evaluate the correlation between the indicators of adiposity, inflammatory markers, and monocyte subtypes in patients with stable CAD.

## 2. Materials and Methods

This was a cross-sectional study conducted using baseline data from a randomized clinical trial including patients >40 years who were diagnosed with CAD by cineangiocoronariography and were clinically stable (more than 60 days without new symptoms, hospitalization, or emergency visit due to cardiovascular disease or new cardiovascular events) [20]. Patients were enrolled from the Interventional Cardiology Service of the Institute of Cardiology of Rio Grande do Sul/University Foundation of Cardiology (IC/FUC, Porto Alegre, Brazil) as well as from public call.

Patients who underwent surgery <90 days previously; had cancer, active infection, or inflammatory diseases; were currently using immunosuppressants; were chronically using anti-inflammatories and immunosuppressive drugs; had grade III obesity (BMI ≥ 40 mg/kg^2^); and were undergoing splenectomy and dialysis were excluded.

This study was approved by the Research Ethics Committee of IC/FUC (No. 534.850), and all individuals agreed to participate by signing the informed consent form.

### 2.1. Data Collection

Data were obtained from medical records and in medical and nutritional consultations; collection was conducted by trained investigators. Sociodemographic, current and cardiac history, and lifestyle variables were evaluated. Patients were categorized as nonsmokers, current smokers, or past smokers (interruption for more than six months [21], alcohol abuse (>30 g/day for men and >15 g/day for women)). The International Physical Activity Questionnaire (IPAQ short version) was used to classify the level of physical activity, and individuals were categorized as inactive, minimally active, or active [22]. Hypertension [23], type 2 diabetes mellitus [24], and dyslipidaemias [25] were defined according to the guidelines, previous diagnosis in medical records, or whether patients were already using medications for their treatment.

### 2.2. Anthropometric Measures and Indicators of Adiposity

Body weight (kg) and height (cm) were measured using an anthropometric electronic scale (Welmy LCD W110H®) and a coupled stadiometer. To measure weight, the patients were asked to be barefoot, empty their pockets, remove adornments, and retain the minimum amount of clothing. By positioning the patient in the centre of the platform, the height was obtained with the patient standing barefoot, with their arms along the sides of their body and their hands facing their thighs.

The waist circumference was measured at the midpoint between the last costal arch and the iliac crest with the patient in the expired supine position. The hip circumference was obtained by identifying the largest diameter of the hip through the major trochanters. The neck circumference was measured with the patient standing with the head positioned in the Frankfurt horizontal plane, measuring the neck perpendicularly to its long axis, at its largest diameter. These indicators were obtained with the aid of an inelastic and flexible tape measure (0 to 143 cm).

The mathematical formulas used for the calculation of traditional and nontraditional indicators of adiposity [5–7] are presented in the Supplementary file (available here). The classification of BMI was determined according to the World Health Organization guidelines.

### 2.3. Laboratory Analysis

Fifteen millilitres of peripheral blood was collected from patients after 12 h of fasting. Serum triglycerides were evaluated by the enzymatic colorimetric method and HDL-cholesterol by immunoprecipitation (Roche modular P Chemistry Analyzer®). The concentration of high-sensitivity C-reactive protein (hs-CRP) was evaluated by the turbidimetry technique (Roche Cobas Integra 400 Plus Chemistry Analyzer®) [26] and fibrinogen levels by the coagulometric method (Sysmex CA-600 systems®) [27].

The cytokines interleukin- (IL-) 2, IL-4, IL-6, IL-10, and interferon-gamma (INF-*γ*) were simultaneously evaluated from plasma aliquots, stored, and frozen at −80°C using the Cytometric Beads Array (CBA) [28] with the detection reagent *T* helper Th1/Th2 (BD Biosciences®, San Diego, CA, USA), according to the manufacturer's instructions. Such analyses were performed in the Laboratory of Clinical Analyses and in the Laboratory of Molecular and Cellular Cardiology of the IC/FUC.

### 2.4. Determination of Subpopulations of Monocytes by Flow Cytometry

The percentage of the different monocyte subtypes was evaluated from peripheral blood that was collected in EDTA tubes using a protocol adapted from Lee et al. [29] and Ulrich et al. [30]. Briefly, blood (300 *µ*l) was incubated with Lysing Buffer™, as instructed by the manufacturer (BD Biosciences®) and then washed with PBS containing 5% foetal bovine serum (Cultilab, SP, Brazil). Subsequently, the cells were incubated with anti-human CD14 FITC (fluorescein isothiocyanate) and CD16 PE-CyTM5 (phycoerythrin cyanine Cy5) antibodies (BD Biosciences®) for 30 minutes.

Immediately after labelling, cells were washed with PBS containing 5% foetal bovine serum, resuspended, and analysed by flow cytometry (FACScanto II, BD Biosciences®). Prior to data acquisition, the sensitivity and overall equipment performance were assessed with CST (Cytometer Setup and Tracking Beads (BD Biosciences®)).

For each sample, at least 8,000 monocyte gate events were acquired and defined according to size (forward side scatter (FSC)) and granularity (side scatter (SSC)) characteristics. Data were analysed with BD software (FACSDiva version 6.1.3, BD Biosciences®) and presented as percentages, as previously described by Ulrich et al. [30]. A figure with a representative gating strategy of the flow cytometric monocyte analysis is included in the Supplementary file (available here).

### 2.5. Sample Analysis and Data Analysis

Sample size calculation was performed using the WinPepi® program for Windows. Considering a Pearson's correlation coefficient (*r*) of 0.33 for the correlation between Mon2 and BMI [31] in patients with high cardiovascular risk, a significance level of 5%, and a power of 90%, it was necessary to evaluate 93 patients.

Analyses were performed using the Statistical Package for Social Sciences, SPSS version 24.0 (IBM Corp., Armonk, NY, USA). Quantitative variables were described as mean ± standard deviation (normal distribution) or median and interquartile range (non-normal distribution). Categorical variables were described as absolute values and percentages. Analysis of variance (ANOVA), Kruskal–Wallis, and Pearson's chi-squared tests were used for comparisons between groups according to the nutritional status classified by BMI. Pearson's correlation coefficient was used for the evaluation of correlations, and adjusted partial correlation was used to evaluate the independent correlations of sex and age. For the correlations, non-normal variables (hs-CRP, LAP, and VAI) were transformed logarithmically. A significance level of 5% was used.

## 3. Results

### 3.1. Characteristics of Participants

In total, 97 patients were evaluated (81.4% men and 18.6% women) with a mean age of 58.0 ± 11.7 years; 94.8% of patients were Caucasian and had a mean educational level of 9.5 ± 3.1 years. Regarding BMI, 13.4% of the patients were classified as having an adequate BMI (BMI, 18.5–24.9 kg/m^2^), 39.2% were overweight (BMI, 25–29.9 kg/m^2^), and 47.4% were classified as obesity (BMI ≥ 30 kg/m^2^).

Table 1 shows the sociodemographic, lifestyle, clinical, anthropometric, and biochemical variables of the participants according to the BMI classification. As expected, the indicators of adiposity were significantly higher in the BMI ≥30 kg/m^2^ category, with the exception of the waist-hip ratio in women (*P*=0.41) and VAI in both sexes. There was no significant difference in relation to the inflammatory markers and percentage of monocyte subtypes according to BMI classification.  Table 2 presents the cardiovascular and drug history of the participants.

### 3.2. Monocytes Subtypes, Inflammatory Markers, and Nutritional Status according to Body Mass Index (BMI)

**Figure 1:**
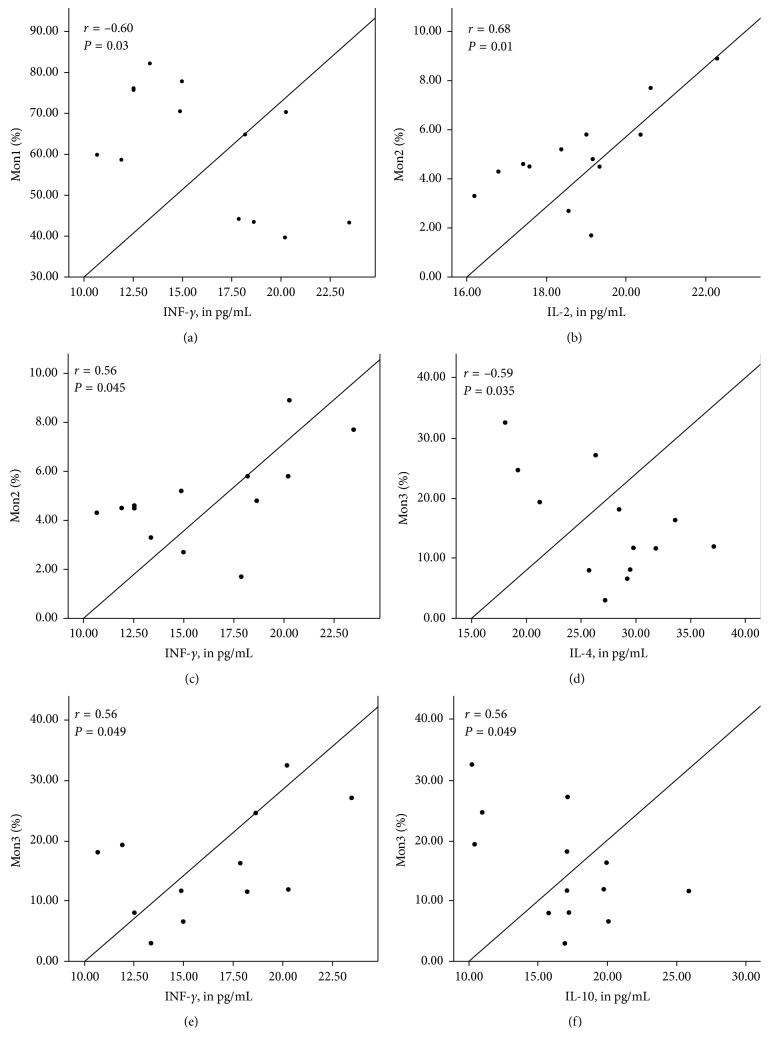
Correlation between percentages of monocyte subgroups (%) and inflammatory markers (pg/mL) in patients with adequate BMI. Mon1 was positively correlated with INF-*γ* (a) and Il-2 (b); Mon2 was positively correlated with INF-*γ* (c) and IL-4 (d); Mon3 was positively correlated with INF-*γ* (e) and negatively correlated with IL-10 (f). (BMI: body mass index).

**Figure 2 fig2:**
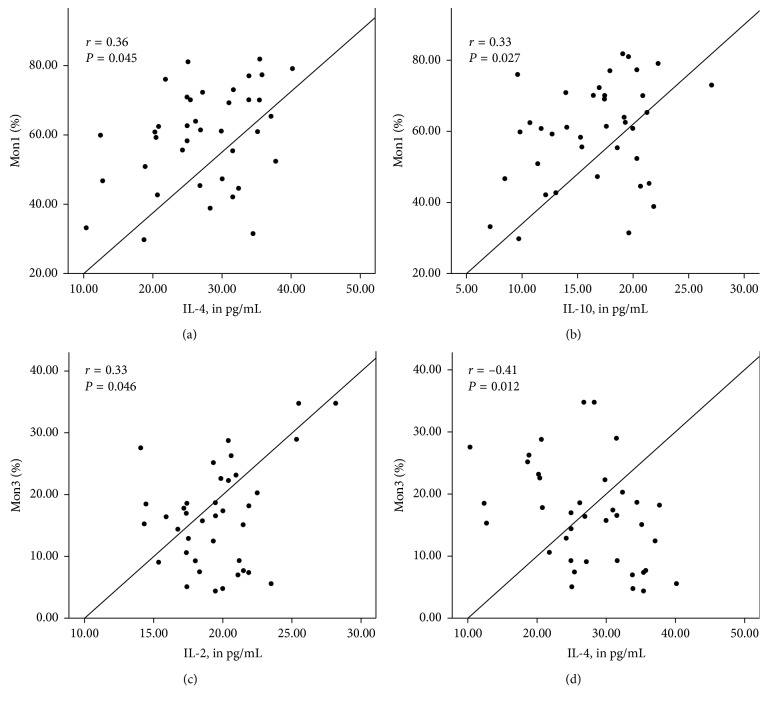
Correlations between percentages of monocyte subgroups (%) and inflammatory markers (pg/mL) in overweight individuals. Mon1 correlated positively with IL-4 (a) and IL-10 (b). Mon3 correlated positively with IL-2 (c) and negatively with IL-14 (d).

**Figure 3:**
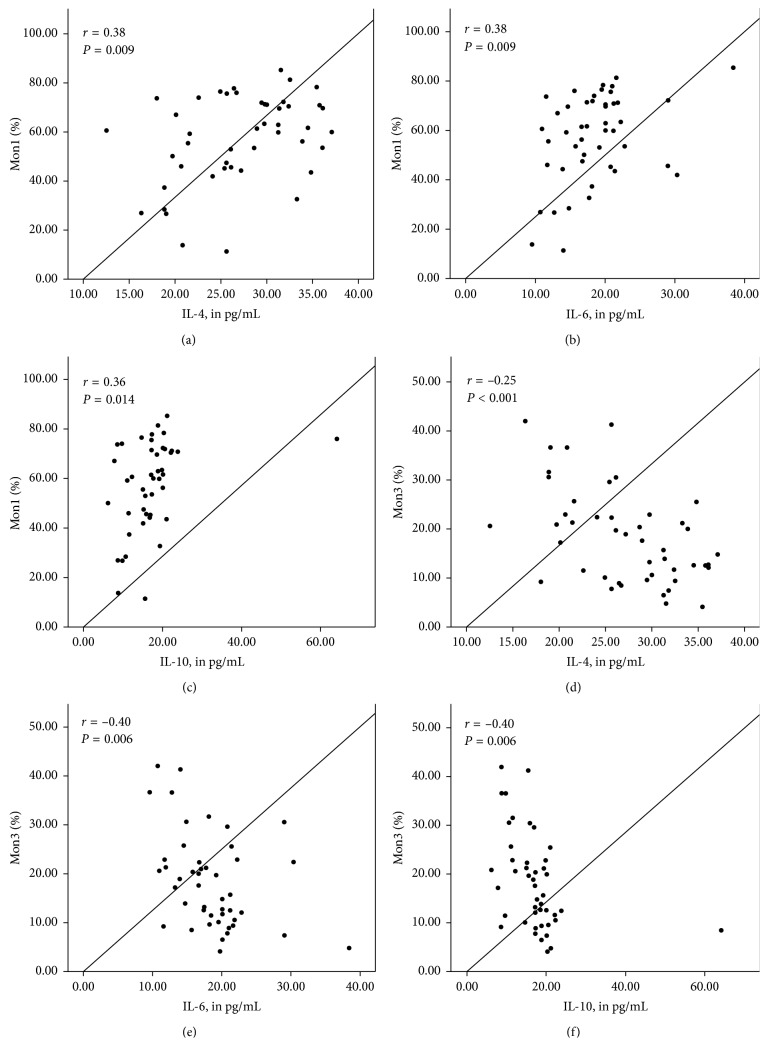
Correlation between the percentage of monocyte subgroups (%) and inflammatory markers (pg/mL) in individuals with obesity. Mon1 correlated positively with IL-4 (a), IL-6 (b), and IL-10 (c). Mon3 correlated negatively with IL-4 (d), IL-6 (e), and IL-10 (f).

Figures 1 [Fig fig2]–3 show the correlation between the percentage of monocyte subtypes and inflammatory markers according to the BMI classification. In patients with adequate BMI (Figure 1), the three monocyte subtypes positively correlated with INF-*γ* (all *P* < 0.05), while the Mon2 positively correlated with IL-2 (*P*=0.01) and Mon3 correlated negatively with IL-10 (*P* < 0.05). In overweight patients (Figure 2), Mon1 correlated positively with IL-4 and IL-10 (*P* < 0.05), while Mon3 correlated positively correlated with IL-2 and negatively with IL-4 (*P* < 0.05). In obese patients (Figure 3), Mon1 presented a positive correlation with IL-4, IL-6, and IL-10 (*P* < 0.05) and Mon3 showed a negative correlation with the same cytokines (*P* < 0.05).

### 3.3. Anthropometric Indicators, Inflammatory Markers, and Nutritional Status according to Body Mass Index (BMI)

The neck circumference correlated negatively with IL-4 concentration in patients with adequate BMI (*r* = −0.60, *P*=0.03). In overweight individuals, the BMI correlated positively with fibrinogen concentration (*r* = 0.37, *P*=0.023). The neck circumference correlated negatively with IFN-*γ* (*r* = −0.33, *P*=0.046) and the LAP correlated negatively with IL-4 concentrations (*r* = −0.41, *P*=0.01). In obese subjects, BMI correlated positively with IL-6 (*r* = 0.44, *P*=0.002), the hip circumference correlated positively with IL-6 (*r* = 0.41, *P*=0.005) and IL-10 (*r* = 0.32, *P*=0.033), and the waist-hip ratio correlated negatively with IL-10 (*r* = −0.36, *P*=0.014).

In overweight individuals, after adjusting for sex and age, the LAP and VAI correlated with IL-4 and fibrinogen and, in obese subjects, the BMI, neck, waist, and hip circumferences, and DAAT correlated positively with IL-6, but only the BMI correlated positively with hs-CRP. There was no correlation between the indicators of adiposity and inflammation among patients with adequate BMI.  Table 3 shows the adjusted partial correlation between the anthropometric indicators that remained related to the respective inflammatory markers in overweight and obese patients, regardless of age and sex.

### 3.4. Anthropometric Indicators, Monocyte Subtypes, and Nutritional Status according to Body Mass Index (BMI)

In patients with adequate BMI, no correlation was observed between the percentage of monocytes and the indicators of adiposity. Among overweight individuals, a positive correlation was observed between the percentage of Mon2 and the hip circumference (*r* = 0.33, *P*=0.046). In obese individuals, the hip circumference correlated positively with the percentage of Mon1 (*r* = 0.39, *P*=0.008) and negatively correlated with the percentage of Mon3 (*r* = −0.33, *P*=0.025).

In overweight patients, after adjustment for sex and age, there was no correlation between the monocyte subtypes and the indicators of adiposity. In obese patients, the hip circumference remained positively correlated with Mon1 (*r* = 0.40, *P*=0.007) and negatively correlated with Mon3 (*r* = −0.35, *P*=0.02).

## 4. Discussion

Obesity is a worldwide epidemic and a cardiovascular risk factor that contributes to a 20% increased risk of acute myocardial infarction [32]. In our study, approximately 87% of the patients showed an excess of adiposity, suggesting that obesity was not appropriately handled in these high-risk patients. We also identified correlations between monocyte subtypes (cells involved in atherogenesis) and inflammatory markers in patients with CAD, regardless of nutritional status, defined by BMI.

Similarly, we observed correlations between traditional and nontraditional indicators of adiposity and inflammation in overweight patients, regardless of factors such as sex and age. Finally, we did not observe correlations between monocyte subtypes and nontraditional indicators of adiposity. This suggests that in patients with CAD, especially those with obesity, the assessment of adiposity through simple and easy-to-obtain measures to identify additional cardiovascular risk is still useful.

Similar to Rothe et al. [33], we found unusual proportions of monocyte subtypes. In their clinical trial, in which the effect of fluvastatin versus placebo on monocytes in patients with hypercholesterolemia was assessed, they observed 55%–60% of Mon1, 25%–35% of Mon2, and 7.6% of Mon3, suggesting that statins alter the counts of these cells.

In obese individuals, Mon1 cells were positively related to inflammatory markers, in agreement with previous findings from experimental studies, in which Gr1 +/Ly6C monocytes (Mon1 in murine) presented a more inflammatory profile in atherosclerosis models [34]. On the other hand, although IL-4 is known to be an anti-inflammatory cytokine [35], its deficiency in mice reduced the development of atherosclerotic plaques [36]. Our results also showed a negative relationship between inflammatory cytokines and Mon3 in obese patients, in line with a more restorative phenotype. Interestingly, INF-*γ*, a cytokine produced by Th1 cells with proatherogenic action [37] and capable of activating monocytes *in vitro* [38], showed a relationship with monocytes only in patients with adequate BMI, suggesting that such patients with CAD present with activation of the adaptive immune response through other mechanisms.

A positive correlation of BMI with IL-6 and hs-CRP was observed in obese patients, even after adjusting for sex and age. On the other hand, new indicators of adiposity were positively related to IL-6 in obese patients and negatively with IL-4 in overweight patients, suggesting that patients with greater visceral fat accumulation have suppressed IL-4. In addition, it can be hypothesized that the use of drugs with metabolic effects, such as statins, can influence the relationships between inflammatory markers and the new indicators that represent the VAT since many of these indexes use markers of lipid profile in their mathematical formulas.

In relation to inflammatory markers, fibrinogen concentrations are known to correlate with TAV (identified using an imaging method) in obese individuals [39] and we were able to identify the same relationship using anthropometric indicators (LAP). It should be considered that we identified a number of waist circumferences and waist-hip ratios above the recommended cutoff points for men and women, even among patients with adequate BMI and overweight patients. This would explain the correlation between LAP and VAI and inflammatory markers in nonobese patients since BMI does not distinguish body fat compartments. Although the measure of neck circumference is associated with a poorer cardiometabolic profile [40], in the current study, it showed a correlation with inflammation only in the univariate analysis, suggesting that factors such as sex, age, and medication use are more important when considering this measure in the assessment of cardiovascular risk. On the other hand, the hip circumference was positively related to Mon1 and negatively to Mon3 in obese patients, even after adjusting for age and sex. Although the value of the isolated hip circumference measurement appears to correlate inversely with cardiovascular risk [41] in men, this correlation has been shown to be weaker [42] and cutoff points according to BMI are yet to be established [43]. Moreover, obese patients were shown to be more active than either overweight patients or those with an adequate BMI. Timmermann et al. [44] observed that regular exercise for 12 weeks was able to reduce CD16+ (Mon2 and Mon3) in women, which may have contributed to this finding. Although, physiologically, the volume of TAV causes a change in the recruitment and migration of monocytes [1], we did not observe a relationship between the indicators of adiposity representing TAV and the monocyte subtypes count.

In the I LIKE HOMe study [31], 622 healthcare workers were recruited during their routine medical check-ups to evaluate the correlation between monocyte heterogeneity, obesity, and subclinical atherosclerosis. The specific association between the monocyte subtype count and the distribution of body fat (assessed by hip circumference and/or waist circumference) was evaluated in a second cohort of 115 subjects and found to have no correlation; however, patients at high cardiovascular risk were excluded from this cohort.

These conflicting results may be explained because although there are data from both *in vitro* [18] and experimental studies [45], there remains a lack of data involving the use of peripheral blood monocyte subtypes and their relationship with inflammatory markers in patients with CAD under pharmacological treatment. Although the evaluation of monocytes and peripheral inflammatory cytokines has greater applicability, such measures do not always reflect their behaviour in tissues. For example, in a study by Bonder et al. [35], IL-4 inhibited the production of IL-10 and IL-12 by isolated peripheral blood monocytes; however, in monocytes of the synovial fluid of patients with rheumatoid arthritis, IL-4 suppressed only IL-12.

Our study has several limitations. Most of the patients were Caucasian men; the number of patients with adequate BMI was small; we did not perform imaging tests as a gold standard method to evaluate the body fat distribution and identify TAV and TAS compartments; our findings do not define causality, considering the cross-sectional design of the study; we did not specifically evaluate the atopic state of the participants; there was no control group including individuals without CAD; and we did not evaluate the important cytokines, monocyte chemoattractant protein-1 (MCP-1), and tumour necrosis factor-alpha (TNF-*α*).

However, the results aim to enrich the knowledge on the behaviour of monocyte subtypes in patients with CAD who are undergoing treatment. Furthermore, we sought to understand the correlation between monocyte subtypes, low-grade inflammation, and different AIs according to the presence or absence of obesity in these individuals. In addition, some previous studies did not follow a standardized methodology for the identification of monocyte subtypes, which was defined more recently in a consensus published in 2016 [14].

## 5. Conclusions

In our study, conducted in patients with stable CAD, we identified correlations between traditional and nontraditional indicators of adiposity, inflammatory markers, and monocyte subtypes, regardless of nutritional status according to BMI. However, this relationship needs to be evaluated further because inherent variables, such as metabolic and hemodynamic changes, as well as drug use, may have an influence on these correlations.

## Figures and Tables

**Table 1:** Baseline clinical characteristics according to body mass index (*n* = 97).

	BMI 18.5 to 24.9 (*n* = 13)	BMI 25.0 to 29.9 (*n* = 38)	BMI ≥ 30.0 (*n* = 46)	*P*value
Age (years)	54.6 ± 18.0	59.5 ± 8.3	57.8 ± 12.1	0.44
Sex				0.90
Male	10 (12.7)	31 (39.2)	38 (48.1)	
Female	3 (16.7)	7 (38.9)	8 (44.4)	
Smoking^1^	9 (14.8)	20 (32.8)	32 (52.5)	0.25
Alcohol abuse	0	1 (25)	3 (75)	0.49
IPAQ				0.54
Active	4 (12.9)	12 (38.7)	15 (48.4)	
Minimally active	4 (10.8)	18 (48.6)	15 (40.5)	
Inactive	5 (17.2)	8 (27.6)	16 (55.2)	
Type 2 diabetes mellitus	3 (15)	8 (40)	9 (45)	0.96
Dyslipidemia	4 (7.3)	24 (43.6)	27 (49.1)	0.12
Hypertension	4 (6.8)	25 (42.4)	30 (50.8)	0.06
BMI (kg/m^2^)	23.8 ± 1.2	27.4 ± 1.4	33.4 ± 3.6	<0.001
WC (cm)				
Male	88.4 ± 6.4	96.6 ± 5.2	110.0 ± 9.2	<0.001
Female	84.3 ± 3.1	92.5 ± 7.5	101.4 ± 5.4	0.002
HC (cm)				
Male	93.9 ± 3.9	99.6 ± 3.7	109.1 ± 8.7	<0.001
Female	96.3 ± 4.2	100.1 ± 6.1	110.1 ± 5.8	0.003
WHR				
Male	0.94 ± 0.06	0.97 ± 0.06	1.00 ± 0.06	0.002
Female	0.88 ± 0.04	0.93 ± 0.06	0.92 ± 0.06	0.41
NC (cm)				
Male	37.4 ± 1.5	39.3 ± 2.2	42.7 ± 3.1	<0.001
Female	33.6 ± 1.7	35.3 ± 2.5	37.2 ± 1.6	0.002
LAP (cm/mmol/l)				
Male	27.67 (21–53)	42.09 (28–71)	80.18 (58–21)	<0.001
Female	30.14 (13–39)	38.55 (35–51)	85.09 (49–113)	0.019
VAI (log)				
Male	3.35 (2.4–4.7)	3.64 (2.7–7.5)	5.18 (3.4–8.3)	0.09
Female	3.44 (1.6–4.6)	4.76 (3.1–6.1)	7.05 (3.3–9.3)	0.16
DAAT (cm^2^)				
Male	170.0 ± 43.8	224.2 ± 34.2	309.9 ± 61.8	<0.001
Female	104.3 ± 14.1	143.4 ± 20.3	179.4 ± 25.0	0.002
IL-2 (pg/mL)	18.8 ± 1.7	19.6 ± 3.1	18.6 ± 1.9	0.19
IL-4 (pg/mL)	27.5 ± 5.5	27.4 ± 7.4	27.2 ± 6.1	0.99
IL-6 (pg/mL)	17.4 ± 3.0	17.7 ± 4.6	18.5 ± 5.5	0.68
IL-10 (pg/mL)	16.8 ± 4.4	16.5 ± 4.6	17.2 ± 8.3	0.90
INF-*γ* (pg/mL)	16.1 ± 3.9	18.3 ± 5.6	17.5 ± 4.0	0.33
hs-CPR (mg/L)	0.10 (0.1–0.4)	0.14 (0.02–0.3)	0.21 (0.1–0.4)	0.81
Fibrinogen (mg/dL)	298.9 ± 69.5	283.8 ± 63.9	284.0 ± 64.1	0.74
Mon1 (%)	62.1 ± 15.0	59.5 ± 14.2	57.3 ± 17.9	0.61
Mon2 (%)	4.9 ± 1.9	5.8 ± 2.2	5.2 ± 2.5	0.39
Mon3 (%)	15.3 ± 8.7	16.5 ± 8.2	18.4 ± 9.6	0.45

Values are presented as mean ± standard deviation, median (interquartile range), or *n* (%). ^1^Current or past smoking. IPAQ: International Physical Activity Questionnaire; BMI: body mass index; WC: waist circumference; HC: hip circumference; WHC: waist-hip circumference; NC: neck circumference; LAP: lipid accumulation product; VAI: visceral adiposity index; DAAT: deep-abdominal-adipose tissue; IL: interleukin; INF-γ: interferon-gamma; hs-CRP: high-sensitivity C-reactive protein; Mon1: classic monocytes; Mon2: intermediate monocytes; Mon3: nonclassic monocytes.

**Table 2:** Baseline characteristics of cardiovascular history and drugs (*n* = 97).

Anterior wall myocardial infarction (%)	22.5
Inferior wall myocardial infarction (%)	21.3
Percutaneous coronary intervention (%)	86.9
Disease in 1 to 2 vessels (%)	65.5
Coronary artery bypass graft (%)	4.8
Ejection fraction (mean ± SD)	56.9 ± 12.4
Killip I (%)	87.8
Family history of CAD (%)	44
Drugs (%)	
Aspirin	86.6
Clopidogrel	51.5
Betablockers	85.5
ACE inhibitor/ARB	79.4
Diuretics	19.6
Statins	81.5

AMI: acute myocardial infarct; CAD: coronary artery disease; ACE: angiotensin-converting enzyme; ARB: angiotensin receptor blocker.

**Table 3:** Partial correlation (*r*)^1^ between inflammatory markers and indicators of obesity in overweight and obese patients.

	BMI 25 to 29.9 (*n* = 38)		BMI ≥ 30.0 (*n* = 46)	
	IL-4	FBR	IL-6	hs-CRP
BMI (kg/m^2^)	−0.09	0.32	0.46^*∗∗*^	0.33^*∗*^
Neck circumference (cm)	−0.18	0.12	0.34^*∗*^	0.20
Waist circumference (cm)	−0.20	0.24	0.34^*∗*^	0.16
Hip circumference (cm)	−0.008	0.07	0.42^*∗∗*^	0.10
WHC	−0.21	0.20	−0.04	0.11
LAP (cm/mmol/l)	−0.45^*∗∗*^	0.35^*∗*^	−0.13	−0.26
VAI (log)	−0.37^*∗*^	0.26	−0.08	−0.26
DAAT (cm^2^)	−0.19	0.26	0.36^*∗*^	0.12

^1^Adjusted for sex and age. BMI: body mass index; WHC: waist-hip circumference; LAP: lipid accumulation product; VAI: visceral adiposity index; DAAT: deep-abdominal-adipose tissue; IL: interleukin; FBR: fibrinogen; hs-CRP: high-sensitivity C-reactive protein. ^*∗*^ *P* < 0.05; ^*∗∗*^ *P* < 0.01.

## Data Availability

The values behind the means, standard deviations and other measures reported used to support the findings of this study are available from the corresponding author upon request.

## Supplementary Materials

Supplementary Materials Table S1: mathematical formulas used for calculation of traditional and nontraditional indicators of adiposity. Figure S1: example of a representative gating strategy of the FACS monocyte analysis of our study. Mon1: classic monocytes; Mon2: intermediate monocytes; Mon3: nonclassic monocytes.
