# Supplementary material for: Correlations between Traditional and Nontraditional Indicators of Adiposity, Inflammation, and Monocyte Subtypes in Patients with Stable Coronary Artery Disease
Source: J Obes. 2019 Jul 3;2019:3139278. doi: 10.1155/2019/3139278 (PMC6637687; doi:10.1155/2019/3139278)
Supplement: Supplementary Materials — Table S1: mathematical formulas used for calculation of traditional and nontraditional indicators of adiposity. Figure S1: example of a representative gating strategy of the FACS monocyte analysis of our study. Mon1: classic monocytes; Mon2: intermediate monocytes; Mon3: nonclassic monocytes. [file 3139278.f1.doc]

**Supplementary Material**

**Table S1.** Mathematical formulas used for calculation of traditional and no-traditional indicators of adiposity.

| Body mass index (BMI, kg/m^2^) | weight (kg)/[height (m)]^2^ |
| --- | --- |
| Waist-to-hip ratio (WHR) | WC/HC |
| Lipid Accumulation Product Index (LAP, cm.mmol.l) | men: (WC – 65) x TG |
|  | women: (WC – 58) x TG |
| Visceral Adiposity Index (VAI, log) | men: WC/[39 + (1.88 x BMI) x (TG/1.03) x (1.31/HDL)] |
|  | women: WC/[36.58 + (1.89 x BMI) x (TG/0.81) x (1.53/HDL)] |
| Deep-Abdominal-Adipose-Tissue Index (DAAT, cm^2^) | men: - 382.9 + [1.09 x weight] + [6.04 x WC] + [- 2.29 x BMI] |
|  | women: - 278 + [- 0.86 x weight] + [5.19 x WC] |

WC: waist circumference, in cm; HC: hip circumference, in cm; TG: serum triglycerides, in mmol/l; HDL: HDL-cholesterol, in mg/dl.


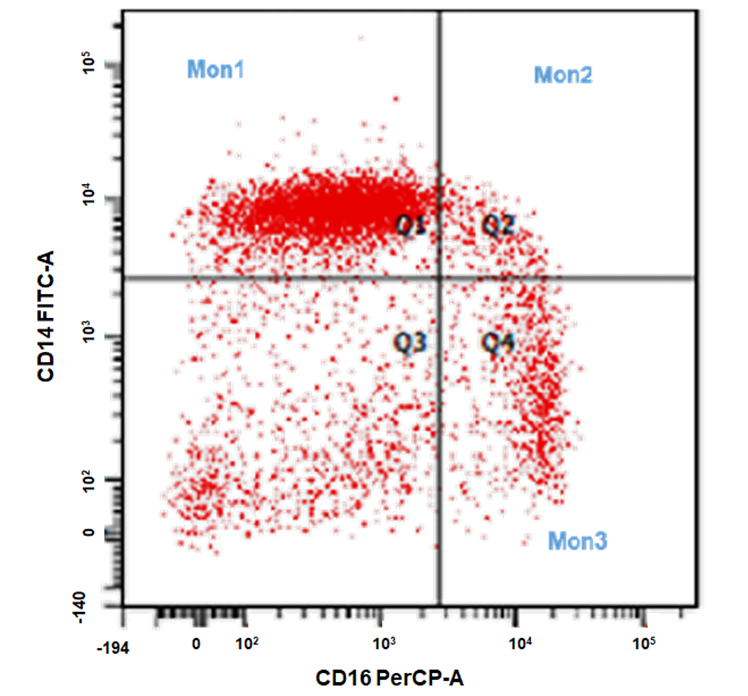


**Figure S1.** Example of a representative gating strategy of the FACS monocyte analysis of our study. Mon1: classic monocytes; Mon2: intermediate monocytes; Mon3: non-classic monocytes.
